# Outcome-Based Quality Control by a Dental Reference Profile of a Population-Based Study (SHIP-0)

**DOI:** 10.1155/2015/794769

**Published:** 2015-07-02

**Authors:** Stefanie Samietz, Andreas Söhnel, Christian Schwahn, Birte Holtfreter, Torsten Mundt, Peter Meisel, Wolfgang Hoffmann, Thomas Kocher, Reiner Biffar

**Affiliations:** ^1^Center of Oral Health, University Medicine Greifswald, Department of Prosthetic Dentistry, Gerodontology and Biomaterials, Rotgerberstraße 8, 17475 Greifswald, Germany; ^2^Center of Oral Health, Department of Restorative Dentistry, Endodontology and Periodontology, Unit of Periodontology, University Medicine Greifswald, Rotgerberstraße 8, 17475 Greifswald, Germany; ^3^Institute of Community Medicine, Section Epidemiology of Health Care and Community Health, University Medicine Greifswald, Ellernholzstraße 1-2, 17487 Greifswald, Germany

## Abstract

*Objectives*. The aim was to develop an instrument for quality control in dental practices. We compared the number of teeth of subjects of the Study of Health in Pomerania (SHIP-0) with those from patients of dental practices. *Methods*. Patients from seven dental practices (*n* = 1,497) were randomly sampled by age strata and gender for a period of two years. Dental status derived from patient files was transformed into practice profiles using age-specific number of teeth as a parameter. Practice profiles were compared with a nomogram, which was based on the age-specific number of teeth of 3,990 SHIP-0 participants regularly visiting the dentist. Further, negative binomial regression models were evaluated to model associations between the number of teeth with age and dental practices, including interactions. *Results*. The practice profiles ranged between the 45th and 95th quantile curves of the reference population SHIP-0. The rate ratios (RR) for the number of missing teeth ranged from 0.37 to 0.67 (*p* < 0.001) between the different dental practices, indicating lower risk for higher numbers of missing teeth in comparison to SHIP-0. *Conclusions*. This study showed considerable differences between dental practices and the reference population of SHIP-0 regarding the pattern of tooth loss and confirms the value of nomograms to compare age-specific numbers of teeth between patients of dental practices and a population-based-study as a tool for quality control. For further analyses, the socioeconomic status of patients and relevant risk factors will be used to adjust for structural differences in order to improve the validity of the comparisons.

## 1. Introduction

Quantitative instruments are an essential part of the decision-making process and have been used in industry as a part of quality management [1–3]. Applying industry-proven models in public health services has been recommended by previous studies [1].

It is assumed that control instruments that evaluate the efficiency and success of health care will become increasingly relevant [4–6]. As a tool for quality management, benchmarking compares an organization's performance, product, or process with similar organizations that are known to be the best [2]. Furthermore, benchmarking allows the identification of areas of improvement. In recent years, outcome control instruments have been used in medicine to document and improve efficiency and success, for example, to record mortality as an outcome of carotid endarterectomy [4], to establish benchmarks for inadvertent perioperative hypothermia [7], and to determine best performance benchmarks for organ transplantation [8].

After the legal introduction of an internal quality management system for contracting dentists (health care providers in the national health insurance system) the quality aspect of services has become increasingly significant [9–11]. Dental practices require standard definitions and observance to assure quality [3, 5, 10, 11]. Evaluation of the efficiency and success of dental health care can be measured by outcome assessments [2]. The patients' immediate interest is in the quality of the outcome, not in the quality of the standardized structure and process [6]. Recently, our working group [2] compared data from dental practices with those from a population-based oral health survey using probing depth, clinical attachment loss, and number of teeth as benchmarking profiles. Because “best dental practice” data are nonexisting, we used the patients' age-specific number of teeth as a surrogate by which we define the quality of dental practice.

We suggest that retaining the maximum number of teeth should be the primary goal of a dental office. As it is not known how many teeth can be retained in daily routine, we used the number of teeth of subjects from a population-based sample as a reference. Because dentists can only facilitate tooth retention in patients who regularly visit a dental office, we restricted the reference population to subjects who had visited their dentist at least once a year for the last two years.

The aim of this investigation was to develop an instrument for quality control in dental practices based on a cross-sectional, population-based study in northeast Germany (Study of Health in Pomerania (SHIP-0)) using the number of teeth as the primary outcome. The main focus was to develop a comprehensive and empirically testable method to measure dental service quality.

## 2. Materials and Methods

### 2.1. Data Collection

The Study of Health in Pomerania (SHIP-0) is a population-based, cross-sectional study in northeast Germany [12, 13]. The population in this area was 212,157, in 1995, and the study sample was selected using the population registries in which all German citizens must be recorded. A total of 7,008 adults aged 20–79 years were sampled, with 292 subjects in each 5-year age stratum. As a result of several reasons (126 had died, 615 had moved away, and five had severe medical problems), 746 subjects were excluded, resulting in the recruitment base of 6,262 inhabitants. The net random sample included 4,308 individuals (68.8%). Details were described elsewhere [12, 14]. The medical and dental examinations took place in two similarly equipped medical/dental facilities in the cities of Greifswald and Stralsund, Germany. The study conformed to the principles of the Declaration of Helsinki as reflected by an a priori approval of the Ethics Committee of the University of Greifswald. Data collection was conducted between October 1997 and May 2001 after written consent was obtained from each participant. Dental examinations were conducted in 3,990 subjects. Also, 298 subjects not visiting their dentist regularly (at least once within the last two years) were excluded. Finally, data from 3,990 20–74-year-old SHIP-0 participants were analyzed (Table 1). Dental examinations were performed by five dentists (alternating daily). Tooth status was determined and the number of teeth was calculated. The maximum number of teeth in this study was 28 (excluding 3rd molars).

### 2.2. Dental Practices

In seven dental practices, data about dental status, age, and gender was taken from routine dental practice records. Patients, who had visited their dentist at least one time a year during the last two years, were selected as the practice sample in predefined months over a period of two years. For each dental practice, a maximum of 20 patients was randomly selected within strata defined by gender and age (5-year strata). All patients were recruited within a predetermined period of two years. Thus, a total of 1,497 subjects aged 20–74 years (with *n* = 197–220 for each practice) were selected. In the youngest and oldest age groups, there were fewer than 20 patients in some dental practices (Table 1). Two practices were specialist practices that were not limited to periodontology (dental practice C and F). Two dental practices were located in the former eastern part of Germany (dental practice A and G). All dentists had a professional experience of more than 15 years.

Cross-sectional data on tooth status were transformed from individual patient files into practice profiles based on number of teeth. The variable number of teeth included the following dental findings: no abnormality detected, filling, inlay, partial crown, crown, and coronally destroyed teeth. The maximum number of teeth in this study was 28 (excluding 3rd molars). The study was approved by the Local Ethics Committee and the data collection was conducted between February 2005 and March 2006.

### 2.3. Statistical Analysis

The age-specific number of teeth was compared between practices and SHIP-0 using two different analytical approaches. In the first approach, the age-specific number of teeth (median and interquartile range) of the patients from different dental practices was analyzed in comparison to the age-specific number of teeth of SHIP subjects using the Mann-Whitney *U* test adjusted for multiple comparisons. Data derived from SHIP-0 were used to generate nomograms of the age-specific number of teeth. Subjects were divided into eleven 5-year strata. For each stratum, the 5th, 25th, 50th, 75th, and 95th quantiles of the number of teeth were calculated and depicted as a nomogram of SHIP-0 data. For the dental practices, only the median number of teeth was calculated and graphically compared within the nomogram.

In the second approach, a negative binomial regression model was used to evaluate the association between the number of missing teeth (dependent variable) and age and dental practice (with SHIP as the reference group). Interactions between age and dental practices were calculated to model potentially differential effect on the number of missing teeth.

Statistical analyses were performed using the commercially available statistical software SPSS version 14.0 for Windows (SPSS Inc., IL, USA) and STATA 8.2 for Windows (College Station, TX, USA).

## 3. Results

### 3.1. Comparison of the Number of Teeth between Dental Practices and SHIP

The number of teeth (median and interquartile range) of patients of the seven practices is presented for comparison with data from the SHIP-0 participants in Table 2. In the older age groups (60–64, 65–69, and 70–74 years), of six practices, there was a large variance among the number of teeth, which is reflected in an interquartile range (IQR) higher than 11 teeth.

For practices A and G (located in the eastern part of Germany) only few age groups were found, for which age-specific numbers of teeth differed significantly from those found in SHIP-0 participants. For practice G, participants older than 60 years presented with the overall lowest median for the number of teeth compared to other practices. For patients of dental practices B, C, D, E, and F the number of teeth differed significantly from that of SHIP for most of the age groups (*p* < 0.05). Particularly in patients of older age (60+ years), practices C, E, and F presented high median levels for the number of teeth. Below the age of 50 there were no pronounced differences in the number of teeth between all practices.

### 3.2. Evaluation of the Dental Nomogram

**Figure 1:**
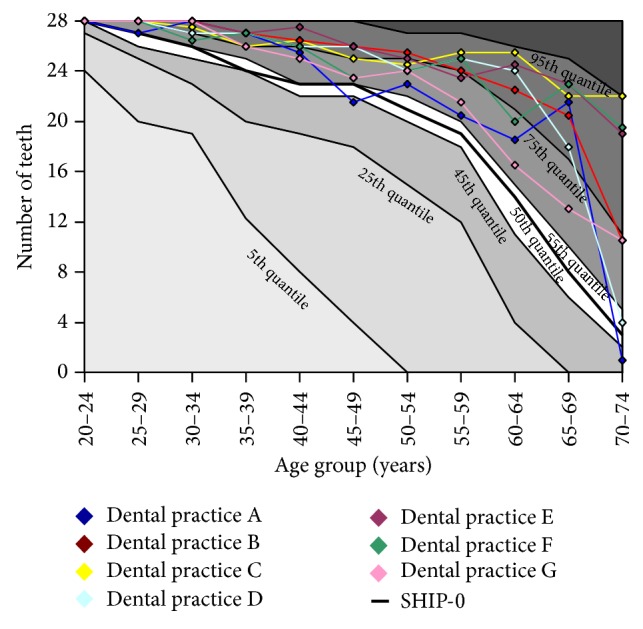
Nomogram for seven dental practices (A to G) in comparison to participants of the Study of Health in Pomerania (SHIP-0). Quantile curves (black solid lines in the background with respective areas between quantile curves colored from light grey to dark grey) were calculated from SHIP-0 and present age-specific quantiles (5th, 25th, 45th, 50th, 55th, 75th, and 95th). For each dental practice, the age-specific median number of teeth is shown.

Data from dental practices were compared to the nomogram (norm profile) of age-specific numbers of teeth derived from SHIP-0. The practice profiles for the patients' number of teeth, that is, the median levels for the number of teeth, were located between the 45th and the 95th quantile curves derived from SHIP-0 (Figure 1). Predominantly, dental practices showed higher median levels for the number of teeth across all age groups as compared to the reference population of SHIP.

Further, we analyzed the extent to which the number of missing teeth differed across age groups and according to dental practice (with SHIP as the reference) using a negative binomial regression model. The impact of the different dental practices (as compared to SHIP) on the number of missing teeth was operationalized as an incidence-rate ratio (IRR) with respect to age and age-practice interactions (Table 3). For age, the IRR was 2.16 (95% CI: 2.10; 2.22; *p* < 0.001) per 5-year age stratum. For dental practices, the IRRs ranged between 0.37 and 0.67 (*p* < 0.001) (reference: SHIP-0). In other words, incidence rates were 0.37 to 0.67 times the incidence rate of SHIP-0.

Dental practice C (periodontology specialist) and dental practice E (general dentist) yielded lowest IRR (IRR = 0.37 and IRR = 0.38, resp.; *p* < 0.001). The two practices located in the eastern part of Germany had highest IRRs (practice A: IRR = 0.67 (0.59; 0.76); practice G: IRR = 0.63 (0.55; 0.73)) but still RRs indicated that expected numbers of missing teeth were significantly lower than in SHIP-0.

Overall, effects of dental practices on the number of missing teeth were modified by age. Incidence-rate ratios for interaction terms between age and dental practices C (IRR = 1.06 (0.92; 1.23)) and F (IRR = 1.05 (0.91; 1.20)), both of which are periodontology specialized practices, were lowest (Table 3). Thus, incidence rates did not depend on age for both dental practices (practice C: *p* = 0.088; practice F: *p* = 0.53). The only two dental practices, for which incidence rates for the number of missing teeth depended on age, were practices B (RR = 1.30 (1.13; 1.50); *p* < 0.001) and D (RR = 1.25 (1.09; 1.43); *p* = 0.001). In other words, incidence rates for both practices were higher in older subjects compared to younger subjects within the same practice.

## 4. Discussion

In this study, individual practice profiles for the number of teeth were compared with a norm profile, which was retrieved from a population-based Study of Health in Pomerania (SHIP-0). As a result, statements on the preventive impact of a specific dental practice can be deduced. Moreover, we found that the number of teeth differed significantly, partly in an age-dependent manner, between patients of single dental practices and SHIP. These discrepancies were partly more pronounced in older age groups.

When receiving dental care, the patients' immediate interests are the outcome quality, the reason a particular treatment must be performed, and how the treatment will influence the outcome, for example, the longevity of the prosthodontic restoration. In light of these factors, practice profiles can be described by a surrogate parameter (number of missing teeth) in order to assess and compare one important dimension of dental practice quality. Instead of using standards for the best outcome, for example, quality indicators or process definitions, we used percentiles/median values based on a representative population-based study as a reference to assess the “performance” of a dental practice. Few studies in health care research have used this method to investigate effect factors as rate ratios [15, 16]. Known risk factors for tooth loss are age and gender [17, 18]. Hence, the choice of age group delineations should be as small as possible when defining a model.

The age-specific number of teeth reflects the quality of a dental practice. Our working group also compared data from dental practices with data from SHIP using probing depth, clinical attachment loss, and the age-specific number of teeth as benchmarking profiles [2] and found a high correlation between attachment loss and the number of teeth [19]. The number of teeth reflects the quality of care provided by a dental practice. The limitations that accompany the clinical evaluation of fillings, endodontics and periodontal parameters notwithstanding data regarding these aspects of dental health are difficult to acquire. The number of teeth is a surrogate parameter for dental outcome [2]. This surrogate parameter is reliable and easily available in dental practice documentation and needs no specific calibration exercise, such as probing depth or clinical attachment loss. The quality of a practice is determined by not only the practice itself but also the patients and the public health system.

The deviation in the age-specific number of teeth of dental practice patients is different from the median absolute deviation value of SHIP-0 participants. Dental practices A and G are located in the former eastern part of Germany and the difference in median values between those two practices and SHIP-0, respectively, was smaller compared to other dental practices. All practices had a preventive effect concerning patient tooth loss in comparison to the norm profile from SHIP-0. Variability was noted among the different practices, which could be explained by different treatment concepts of the individual dentists and different patient clienteles. These values are characterized by the dentists' specialization (e.g., periodontology). The outcome quality of a dental practice is dependent on many parameters, including a certain selection of a patient's properties [20], the utilization of dental treatment and prevention [21], and the individual risk profile of each patient.

A limitation of this study was that no other confounders for tooth loss could be evaluated (e.g., smoking and socioeconomic status) because data were taken out of the patient files. Furthermore, this tool is not applicable to specialty practices such as oral surgeons or implantologists or to practices that are restricted to acute pain-relief dentistry. Our results support the use of this study for family dentists. However, this method is principally adaptable to groups of special practices via the development of a special nomogram for each group.

The sociological conditions and requirements that determine the social class structure differ in different parts of Germany [22]. Education is a key factor influencing awareness of health and illness [23, 24]. Previous studies have shown that school education [25, 26], social marital status [27], oral health behavior, and other socioeconomic factors [28] have a significant influence on tooth loss. The influence of health care on dental outcomes has been described in previous investigations [10, 11, 29, 30]. This study provides a conceptual basis for a more comprehensive study of tooth loss that simultaneously considers (a) socioeconomical profiles, (b) risk factors for tooth loss (e.g., smoking and oral hygiene), (c) other sociodemographic factors (e.g., school education), and (d) the quality of health care provided by dental practices to allow a risk-structured adjusted comparison of profiles among dental practices (patient-related risk profiles) in Germany to nomograms based on SHIP-0.

Furthermore, the utilization and accessibility of medical services have been considered as determining factors of tooth loss [31]. The influence of these factors becomes clear particularly when considering the prevalence of edentulous patients, because this suggests the assumption that edentulous patients do not visit dental practices as frequently as dentate patients in the older age groups. In addition, the evaluation of dental practice characteristics is useful to describe the different practices (e.g., location, recall system, specialization, and professional experience). However, these characteristics provide a limited reflection of practice quality because there is a lack of available data on the number of patients using the prophylaxis offered, the number of dropouts over time, and the number of patients who visit the practice only sporadically [32, 33].

These limitations notwithstanding several strengths of our study merit consideration. We used a large population-based sample to generate a norm profile, which allows for a high degree of generalizability. Furthermore, the randomized sampling design of the patients who visited the practices of their dentists in different months for a period of two years should allow for a representative sampling of the patients of these practices. Studies have shown that 77.0% of the German population visits a dentist at least once a year [21]. Furthermore, the utilization of dentists was confirmed to decrease steadily with increasing age. Population groups with the highest illness risk are often observed to have a low self-help potential [34]. Hence, our practice samples likely represent a positive selection of patient intrinsic characteristic (selection bias) because these patients are similar with respect to health consciousness and self-management.

The advantage of using a variable such as the age-specific number of teeth is that it can quickly be compared with data from epidemiological studies (e.g., the German Oral Health Study and the National Health and Nutrition Examination Survey (NHANES)) and can be implemented in quality management tools with multivariate analyses that include risk factors. Furthermore, the expansion of standard profiles using other international population-based data is useful for international comparison and validation of the described method.

For future application in dentistry, the evaluation proposed by the current study of routine data in dental practices could be simply applied by using data administration software systems, which are used in every dental practice for documentation and management. This instrument could be a useful tool for collecting epidemiological dental data using standard documentation as a source. Evidence that quality improvement strategies can enhance the processes and outcomes of medical care and that self-assessment is a powerful tool for quality assurance in medical care has been shown in previous studies [29, 30].

This study confirms the value of nomograms to compare age-specific numbers of teeth between patients in dental practices and participants of a population-based study as a tool for quality management. The visualization of the outcome using nomograms can help dentists in strategy refinement and decision making in their everyday dental treatment. This instrument is a powerful tool for self-assessment of quality assurance in dental treatment strategies as part of a systematic process for structural learning and continuous improvement. With quality management, the transparency of dentists' performance is increased in dental practice and quality-driven elements are enhanced in the competition. Another useful future application of nomograms could be the evaluation of study populations in clinical studies in association with population-based cohorts, which would validate the findings and allow higher degrees of generalizability.

## 5. Conclusion

This instrument makes the quality of a dental practice more transparent and can help in the determination of a practice profile, potentially yielding benefits for the dentist, his or her patients, health insurance companies, and public health research. Furthermore, this instrument is a powerful tool in self-assessment for quality assurance in dental treatment strategies as part of a systematic process for structural learning and continuous improvement. Ideally, this tool will be useful for providing feedback to practices with which to reflect on their performance.

## Figures and Tables

**Table 1:** Distribution according to age for SHIP-0 participants and patients from seven dental practices.

Age group (years)	SHIP-0		Dental practice A		Dental practice B		Dental practice C		Dental practice D		Dental practice E		Dental practice F		Dental practice G	
	*n*	%	*n*	%	*n*	%	*n*	%	*n*	%	*n*	%	*n*	%	*n*	%
20–24	268	6.7	20	9.3	20	9.1	20	9.3	20	9.2	20	9.1	18	8.5	11	5.6
25–29	324	8.1	20	9.3	19	8.7	19	8.8	20	9.2	20	9.1	18	8.5	14	7.1
30–34	375	9.4	20	9.3	20	9.1	20	9.3	20	9.2	20	9.1	20	9.4	15	7.6
35–39	385	9.6	20	9.3	20	9.1	20	9.3	20	9.2	20	9.1	20	9.4	17	8.6
40–44	370	9.3	20	9.3	20	9.1	20	9.3	20	9.2	20	9.1	20	9.4	20	10.2
45–49	378	9.5	20	9.3	20	9.1	20	9.3	20	9.2	20	9.1	20	9.4	20	10.2
50–54	366	9.2	20	9.3	20	9.1	20	9.3	20	9.2	20	9.1	20	9.4	20	10.2
55–59	423	10.6	20	9.3	20	9.1	20	9.3	20	9.2	20	9.1	20	9.4	20	10.2
60–64	419	10.5	20	9.3	20	9.1	20	9.3	20	9.2	20	9.1	20	9.4	20	10.2
65–69	370	9.3	18	8.4	20	9.1	20	9.3	20	9.2	20	9.1	20	9.4	20	10.2
70–74	312	7.8	17	7.9	20	9.1	17	7.9	18	8.3	20	9.1	16	7.8	20	10.2

Total	3990	100	215	100	219	100	216	100	218	100	220	100	212	100	197	100

**Table 2:** Number of teeth (median and interquartile range) for patients from seven dental practices.

Age group (years)	Number of teeth (median and IQR)							
	Dental practice A (*N* = 215)	Dental practice B (*N* = 219)	Dental practice C (*N* = 216)	Dental practice D (*N* = 218)	Dental practice E (*N* = 220)	Dental practice F (*N* = 212)	Dental practice G (*N* = 197)	SHIP-0 (*N* = 3990)
20–24	28 ± 2	28 ± 1	28 ± 1	28 ± 1	28 ± 0	28 ± 0	28 ± 1	28 ± 2
25–29	27 ± 2	28 ± 1^*∗*^	28 ± 1	28 ± 1^*∗*^	28 ± 1^*∗*^	28 ± 2	28 ± 1	27 ± 4
30–34	28 ± 2	28 ± 1^*∗*^	28 ± 1^*∗*^	27 ± 3^*∗*^	28 ± 2^*∗*^	27 ± 4	28 ± 2^*∗*^	25 ± 4
35–39	27 ± 5^*∗*^	27 ± 2^*∗*^	26 ± 5^*∗*^	27 ± 3^*∗*^	27 ± 2^*∗*^	27 ± 5^*∗*^	26 ± 5	24 ± 5
40–44	26 ± 4^*∗*^	27 ± 4^*∗*^	27 ± 4^*∗*^	26 ± 4^*∗*^	28 ± 2^*∗*^	26 ± 4^*∗*^	25 ± 5	23 ± 7
45–49	22 ± 4	26 ± 5	25 ± 3^*∗*^	26 ± 6^*∗*^	26 ± 4^*∗*^	24 ± 6	24 ± 8	23 ± 7
50–54	23 ± 7	26 ± 9^*∗*^	25 ± 4^*∗*^	24 ± 8	25 ± 5^*∗*^	24 ± 4^*∗*^	24 ± 9	21 ± 9
55–59	21 ± 11	24 ± 9^*∗*^	26 ± 7^*∗*^	25 ± 8^*∗*^	24 ± 5^*∗*^	25 ± 6^*∗*^	22 ± 8	19 ± 12
60–64	19 ± 18	23 ± 15^*∗*^	26 ± 7^*∗*^	24 ± 12^*∗*^	25 ± 6^*∗*^	20 ± 12^*∗*^	17 ± 17	14 ± 17
65–69	22 ± 10^*∗*^	21 ± 17^*∗*^	22 ± 7^*∗*^	18 ± 11^*∗*^	23 ± 12^*∗*^	23 ± 14^*∗*^	13 ± 18	8 ± 17
70–74	1 ± 14	11 ± 19^*∗*^	22 ± 11^*∗*^	4 ± 21^*∗*^	19 ± 14^*∗*^	20 ± 10^*∗*^	11 ± 21^*∗*^	3 ± 11

Mann-Whitney *U* tests comparing the number of teeth between each dental practice and SHIP-0. *p* values were adjusted according to Bonferroni for multiple comparison; ^*∗*^ *p*_adj_ < 0.0045.

**Table 3:** Multivariable negative binomial regression model evaluating effects of dental practice and age (including interaction between both) on the number of missing teeth (dependent variable).

	IRR (95% CI)	*p* value
Age	2.16 (2.10; 2.22)	<0.001
Dental practice (reference SHIP)		
Dental practice A	0.67 (0.59; 0.76)	<0.001
Dental practice B	0.43 (0.38; 0.50)	<0.001
Dental practice C	0.37 (0.32; 0.43)	<0.001
Dental practice D	0.47 (0.41; 0.54)	<0.001
Dental practice E	0.38 (0.33; 0.43)	<0.001
Dental practice F	0.47 (0.41; 0.53)	<0.001
Dental practice G	0.63 (0.55; 0.73)	<0.001

Interaction terms between age and dental practice		
Age × dental practice A	1.12 (0.98; 1.28)	0.088
Age × dental practice B	1.30 (1.13; 1.50)	<0.001
Age × dental practice C	1.06 (0.92; 1.23)	0.428
Age × dental practice D	1.25 (1.09; 1.43)	0.001
Age × dental practice E	1.15 (1.00; 1.32)	0.053
Age × dental practice F	1.05 (0.91; 1.20)	0.530
Age × dental practice G	1.16 (1.00; 1.38)	0.053

Age was *z*-standardized and modelled continuously. IRR: incidence-rate ratio; CI: confidence interval. Pseudo *R* ^2^ = 0.1.
